# Identification and characterization of an R-Smad homologue (*Hco*-DAF-8) from *Haemonchus contortus*

**DOI:** 10.1186/s13071-020-04034-0

**Published:** 2020-04-03

**Authors:** Fang-Fang Li, Robin B. Gasser, Feng Liu, Jia-Nan Shan, Wen-Da Di, Li He, Cai-Xian Zhou, Chun-Qun Wang, Rui Fang, Min Hu

**Affiliations:** 1 State Key Laboratory of Agricultural Microbiology, Key Laboratory for the Development of Veterinary Products, Ministry of Agriculture, College of Veterinary Medicine, Huazhong Agricultural University, Wuhan, 430070 Hubei China; 2 Melbourne Veterinary School, Department of Veterinary Biosciences, Faculty of Veterinary and Agricultural Sciences, The University of Melbourne, Parkville, Victoria 3010 Australia

**Keywords:** *Haemonchus contortus*, R-Smads, TGF-β signalling pathway, Development, Gene rescue, Specific inhibitor of human Smad3 (SIS3)

## Abstract

**Background:**

Smad proteins are essential cellular mediators within the transforming growth factor-β (TGF-β) superfamily. They directly transmit incoming signals from the cell surface receptors to the nucleus. In spite of their functional importance, almost nothing is known about Smad proteins in parasitic nematodes including *Haemonchus contortus*, an important blood-sucking nematode of small ruminants.

**Methods:**

Based on genomic and transcriptome data for *H. contortus* and using bioinformatics methods, a Smad homologue (called *Hco*-*daf-8*) was inferred from *H*. *contortus* and the structural characteristics of this gene and its encoded protein *Hco*-DAF-8 established. Using real-time PCR and immunofluorescence assays, temporal transcriptional and spatial expression profiles of *Hco*-*daf-8* were studied. Gene rescue in *Caenorhabditis elegans* was then applied to assess the function of *Hco-daf-8* and a specific inhibitor of human Smad3 (called SIS3) was employed to evaluate the roles of *Hco*-DAF-8 in *H. contortus* development.

**Results:**

The features of *Hco-*DAF-8 (502 amino acids), including conserved R-Smad domains and residues of the L3-loop that determine pathway specificity, are consistent with a TGF-β type I receptor-activated R-Smad. The *Hco-daf-8* gene was transcribed in all developmental stages of *H. contortus* studied, with a higher level of transcription in the fourth-stage larval (L4) females and the highest level in adult males. *Hco*-DAF-8 was expressed in the platymyarian muscular cells, intestine and reproductive system of adult stages. Gene rescue experiments showed that *Hco-daf-8* was able to partially rescue gene function in a *daf-8* deficient mutant strain of *C. elegans*, leading to a resumption of normal development. In *H. contortus*, SIS3 was shown to affect *H. contortus* development from the exsheathed third-stage larvae (L3s) to L4s *in vitro*.

**Conclusions:**

These findings suggest that *Hco*-DAF-8, encoded by the gene *Hco-daf-8*, is an important cellular mediator of *H. contortus* development *via* the TGF-β signalling pathway. They provide a basis for future explorations of *Hco*-DAF-8 and associated pathways in *H. contortus* and other important parasitic nematodes.
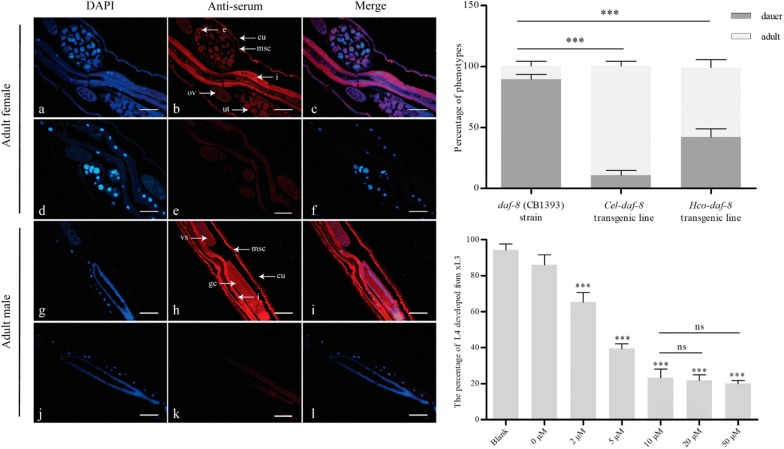

## Background

The transforming growth factor-β (TGF-β) signalling pathway regulates the growth, development and differentiation of cells in diverse organisms including humans, mice, flies and worms [1, 2]. In this pathway, signalling is initiated when the ligand induces the assembly of a heteromeric complex of type II and type I receptors. Upon activation of type I receptors, signalling from cell-surface receptors to the nucleus is mediated by Smad proteins.

Based on their functions, Smad proteins are classified into three subclasses, i.e. receptor-regulated Smads (R-Smads), common-partner Smads (Co-Smads) and inhibitory Smads (I-Smads) [3]. R-Smads are direct substrates of type I receptors, represented by TGF-β/Activin-activated R-Smads and BMP*-*activated R-Smads. The former molecules are specifically activated by activin/nodal and TGF-β type I receptors, such as *Hs*-Smads 2 and 3; the latter molecules are activated by BMP (bone morphogenetic protein) type I receptors, including *Hs*-Smads 1, 5 and 8 [4]. Following phosphorylation by a type I receptor, R-Smads interact with Co-Smads to form a complex, which is then translocated into the nucleus to regulate the transcription of target genes. In these processes, R-Smads usually play a central role in maintaining specificity in the TGF-β signalling pathway by functioning both as a substrate of the type I receptor and as an usher for Co-Smad [5]. In addition, R-Smads are regulated by inhibitory Smads, which prevent the phosphorylation and/or nuclear translocation of R-Smads [6].

In *Caenorhabditis elegans*, the TGF-β signalling pathway (*daf-7* pathway) functions to regulate dauer formation *via* the R-Smads *Cel-*DAF-8 and *Cel-*DAF-14 [7]. In favourable environments, the *daf-7* signal is received by a heterotetrameric receptor composed of two DAF-1 type I receptor and two DAF-4 type II receptor subunits on the cell surface. The DAF-4 type II receptor first phosphorylates the DAF-1 type I receptor, which then propagates the signal through phosphorylation of the R-Smad proteins *Cel*-DAF-8 and *Cel*-DAF-14. Activated R-Smads complexes are translocated into the nucleus and inhibit the functions of DAF-3/Co-Smad and DAF-5, regulating the transcription of genes involved in normal larval development [8]. Under harsh environmental conditions the *daf-7* pathway is inactive, allowing downstream genes such as *daf-3* and *daf-5* to promote dauer formation [9]. *Cel-*DAF-3 can repress the transcription of *Cel*-DAF-8, which drives a negative feedback loop of this pathway [10]. In *C. elegans*, R-Smads *Cel*-DAF-8 and *Cel*-DAF-14 are co-expressed in a subset of neurons, the intestine and excretory cells, and, interestingly, have partial functional redundancy of one another [10, 11].

In the parasite nematode *H. contortus*, recent studies identified some members of the TGF-β signalling pathway including TGF-β type I receptor [12], TGF-β type II receptor [13] and Co-Smad [14] and evaluated their important roles in larval development from the exsheathed third-stage larvae (L3s) to L4s *in intro*. As there is no information available on these R-Smads for parasitic nematodes of clade V [15], we took the opportunity of exploring the structure and function of the homologue of *Cel-*DAF-8 in *H. contortus* (barber’s pole worm), one of the economically most important parasites of small ruminants worldwide [16].

Herein, a TGF-β-activated R-Smad gene from *H. contortus*, called *Hco-daf-8* was isolated and characterised. The transcription profile of the *Hco-daf-8* gene in distinct developmental stages of *H. contortus* and protein localisation in adults were investigated. Gene rescue experiment showed that *Hco-daf-8* was able to partially rescue the dauer phenotype of *daf-8*-deficient mutant strain of *C. elegans*, and a Smad3-specific inhibitor SIS3 was shown to affect *H. contortus* development from L3s to L4s *in vitro*.

## Methods

### *Caenorhabditis elegans* and *H. contortus* strains, and their maintenance

*Caenorhabditis elegans* strains were purchased from the *Caenorhabditis* Genetic Center (CGC) (University of Minnesota, USA) and cultured with *Escherichia coli* OP50 as a food source on nematode growth medium (NGM) using standard procedures [17], unless otherwise stated. Worm strains used were N_2_ wild-type and a *daf-8* mutant (CB1393), the latter of which is temperature-sensitive and has a dauer phenotype at 25 °C. *Haemonchus contortus* (Haecon 5 strain) employed here was maintained by serial passages in experimental goats (raised helminth-free; 14 weeks of age); kids were infected orally with 7000 third-stage (infective) larvae (iL3s). Eggs isolated from the faeces by sucrose flotation [18] were cultured in tissue culture flasks at 28 °C in a nutritive medium (0.1 ml/ml of culture of 1× Earle’s balanced salt solution (EBSS, Sigma-Aldrich) and 0.5% (w/v) of yeast extract) [19]. First-stage larvae (L1s), second-stage larvae (L2s) and iL3s were obtained from eggs cultured at 28 °C (constant) for 1, 4 and 7 days, respectively [20]. Fourth-stage larvae (L4s; both sexes) and adults (both sexes) were collected from the abomasa of infected goats, euthanased 8 and 30 days, respectively, following oral infection with iL3s [20]. Males and females of L4s and adults were separated as described previously [21].

### DNA and RNA preparation

Genomic DNA was extracted from mixed developmental stages of *C. elegans* using an EasyPure Genomic DNA kit (TransGen Biotech, Beijing, China). Genomic DNA was also isolated from adult *H. contortus* (both sexes) by mini-column (Wizard® Clean-Up; Promega, Beijing, China) purification following small-scale SDS/proteinase K digestion [22]. DNA samples were stored at − 20 °C. Total RNA was isolated using the TRIpure reagent (Aidlab, Beijing, China) as recommended by the manufacturer. RNA quality and yields were verified by electrophoretic analysis and spectrophotometry (NanoDrop Technologies, Wilmington, USA), respectively. First-strand cDNA synthesis was performed using the extracted RNA and PrimeScript™ RT reagent kit with DNA Eraser (Takara, Beijing, China). RNA and cDNA samples were stored at − 80 °C.

### Identification of the *C. elegans* DAF-8 homologue in *H. contortus*

The *Cel-daf-8* gene sequence was obtained from WormBase (WS271; code R05D11.1), and an homologue of this gene was identified by searching the *C. elegans* protein sequence against the non-redundant (nr) protein sequence database using the protein BLAST from the National Center for Biotechnology Information (NCBI) (http://www.ncbi.nlm.nih.gov/BLAST). Homologues of the *Cel-*DAF-8 protein were inferred from the *H. contortus* genomes (PRJEB506 and PRJNA205202) using BLAST/BLAT (https://wormbase.org/tools/blast_blat) [23].

### Isolation of *Hco-daf-8* genomic sequence and coding DNA sequence (CDS)

Using genomic and transcriptomic data sets for *H. contortus* [16, 24], the sequences of the *Hco-daf-8* coding region and full-length genomic region were retrieved (GenBank: HF958885.1). The coding region of *Hco-daf-8* was PCR-amplified from cDNA with primer pair Hco-daf-8-F and Hco-daf-8-R (Additional file 1: Table S1) using the following cycling conditions: 98 °C for 5 min; then 98 °C for 10 s, 55 °C for 5 s, 72 °C for 90 s for 35 cycles; and a final extension step at 72 °C for 5 min. The full-length genomic sequence of *Hco-daf-8* was PCR-amplified from *H. contortus* genomic DNA using the same primer pair and conditions as described above, except that the extension time was 3 min, instead of 90 s. These two PCR products were individually inserted into pTOPO-Blunt vector (CV17; Aidlab) and directly sequenced in both directions (*via* TsingKe Biological Technology, Wuhan, China).

### Bioinformatic and phylogenetic analyses

Nucleotide (nt) sequences and amino acid (aa) sequences were assembled and aligned using the programs BLASTx and Clustal W [25]. Briefly, the sequence of *Hco-daf-8* was compared with sequences in non-redundant databases using the BLASTx from the National Center for Biotechnology Information (NCBI) (http://www.ncbi.nlm.nih.gov/BLAST) to confirm the identity of cloned genes. Each cDNA sequence was conceptually translated into predicted amino acid sequence using DNAstar software (http://www.dnastar.com). To determine the sequence characteristics of *Hco*-DAF-8, amino acid sequence comparison was performed. The predicted amino acid sequence of *Hco*-DAF-8 was aligned with two R-Smads (*Cel*-DAF-8 and *Cel*-DAF-14) involved in the DAF-7 signalling pathway in *C. elegans* and a panel of reference sequences: TGF-β/Activin-activated R-Smads (*Hs-*Smad2, *Hs*-Smad3 of *Homo sapiens*; *Dm-*Smox of *Drosophila melanogaster*; and *Dar*-MAD of *Danio rerio*) and BMP*-*activated R-Smads (*Hs-*Smad1, *Hs*-Smad5 of *H. sapiens*; *Dm*-MAD of *D. melanogaster*; and *Mm-*MAD1 of *Mus musculus*) (Additional file 1: Table S2) using the program Clustal W [25]. Graphic view was conducted using BioEdit software (https://bioedit.software.informer.com). Sequence identity was assessed using Clustal Omega (https://www.ebi.ac.uk/Tools/msa/clustalo/). Protein motifs of *Hco*-DAF-8 were identified by scanning the databases Pfam (www.sanger.ac.uk/Software/Pfam) and PROSITE (http://prosite.expasy.org/). Exon and intron boundaries in DNA sequences were inferred using the software MAFFT (https://mafft.cbrc.jp/alignment/server/) and the “GT-AG” rule [26]. Nuclear localisation signal (NLS) sequence was predicted using the program cNLS Mapper [27].

The alignment of *Hco-*DAF-8 with selected references including *Cel-*DAF-8 and its homologues (*Cbn*-DAF-8 and *Cjp*-DAF-8), *Cel-*DAF-14 and its homologues (*Cbr*-DAF-14 and *Cjp*-DAF-14), *Cel-*SMA-2 and its homologues (*Cbr*-SMA-2; *Ovo*-SMA-2; *Hco*-SMA-2), *Cel-*SMA-3 and *Cbr*-SMA-3, as well as four R-Smads from *Homo sapiens* and Smox from *D. melanogaster* were subjected to phylogenetic analysis. An inhibitory-Smad protein of *D. melanogaster* (*Dm-*DAD) was used as an outgroup (Additional file 1: Table S2). Phylogenetic analyses were conducted using the neighbour-joining (NJ), minimum evaluation (ME) and maximum likelihood (ML) methods employing the Jones–Taylor–Thornton (JTT) model [28]. Confidence limits were assessed using a bootstrap procedure, with 1000 bootstrap replications for NJ and default settings (in MEGA7; [28]) for the other methods. A 50% cut-off value was implemented for the consensus tree.

### Assessing transcript abundance using real-time PCR

Transcription of *Hco-daf-8* was examined in each of the six developmental stages (eggs, L1s, L2s, iL3s, L4s and adults) and both sexes (males and females) of *H. contortus* (Haecon-5 strain) by real-time PCR using the primers rtHco-daf-8-F and rtHco-daf-8-R (Additional file 1: Table S1). RNA was isolated separately from eggs, L1s, L2s and iL3s (10,000 of each stage) and female L4, male L4 (five of each), female adults or male adults (three of each) using TRIpure Trizol (Aidlab) according to the manufacturer’s protocol. An equal amount (1 μg) of RNA from each stage was used to synthesise the first-strand cDNA by random primer using PrimeScript™ RT reagent Kit with gDNA Eraser (Takara), respectively. The RT-PCR (10 μl) was performed using the TB Green™ Premix Ex Taq™ II (Tli RNaseH Plus) (Takara, No. RR820A) in a thermal cycler (AII A7; Bio-Rad, Berkeley, USA) under the following protocol: 50 °C for 2 min and 95 °C for 30 s for the first cycle, followed by 95 °C for 15 s, 60 °C for 15 s and 72 °C for 20 s for 40 cycles. Each sample was tested in triplicate, employing a β-tubulin 8–9 gene (GenBank: M76493) as a reference gene (using specific primers Tubulin-F and Tubulin-R; Additional file 1: Table S1) [29]. The mean quantification cycle (Cq) values were subjected to compare the relative quantities with egg (egg = 1) using the 2^−∆∆Cq^ method [30]. This assay was repeated three times. Statistical analysis was carried out using one-way ANOVA in GraghPad Prism 6 (https://www.graphpad.com/support/faqid/1745/). *P*-values were calculated using the Tukey’s *post-hoc* test; values of < 0.05 were considered statistically significant.

### Production of polyclonal antibody against recombinant *Hco*-DAF-8 and immunoblot analysis

The truncated cDNA fragment of *Hco-*DAF-8 coding for 251 aa was PCR-amplified using the primer pair rHco-DAF-8-F/R (Additional file 1: Table S1) and then cloned into the prokaryotic expression vector pGEX-4T, to construct the prokaryotic expression plasmid pGEX-4T-*Hco-*DAF-8. The insert was sequenced, and the recombinant plasmid was then transferred to BL21 (DE3) cells. Recombinant *Hco-*DAF-8 (r*Hco-*DAF-8) expression was induced by 1 mM isopropyl β-D-1-thiogalactopyranoside (IPTG) at 16 °C overnight. Expressed proteins were purified from culture supernatants using the glutathione sepharose™ 4B column system (GE Healthcare, Pittsburgh USA) and then used to immunise rabbits to produce anti-r*Hco*-DAF-8 polyclonal antibody. Briefly, recombinant protein r*Hco-*DAF-8 (500 μg) was administered three times subcutaneously 14 days apart and once intravenously (100 μg) 6 days prior to euthanasia using pentobarbital. Serum was collected and affinity purified over a Protein A-sepharose column (Thermo Fisher Scientific, Waltham, USA) and quantified [31]. Pre-immune rabbit serum (negative control) was processed in the same manner. All sera were stored at − 80 °C.

Affinity-purified antibody against r*Hco*-DAF-8 was used for immunofluorescence and immunoblot analyses. For immunoblot, proteins extracted from adult male or female *H. contortus* worms using a Total Protein Extraction Kit (BestBio, Shanghai, China) were electrophoresed in 12% sodium dodecyl sulfate (SDS)-polyacrylamide gel and transferred to an Immobilon®-PSQ Transfer Membrane (Merck Millipore Ltd., Darmstadt, Germany). After being blocked with 1% (w/v) BSA (BioFROXX, Guangzhou, China) in phosphate-buffered saline (PBS, pH 7.4) containing 20% Tween-20 (PBST) for 6 h at 4 °C, the membranes were incubated in the primary antibody against r*Hco-*DAF-8 (diluted 1:1000 in PBST) overnight at 4 °C, followed by 5 min × 6 washes in PBST and subsequent incubation with the goat anti-rabbit IgG secondary antibody conjugated with horse radish peroxidase (Beyotime Biotechnology, Shanghai, China) diluted 1:1000 in PBST for 2 h at 37 °C. After five washes of the membranes, immunodetection was carried out by chemiluminescence (WesternBright ECL kit, cat. no. K-12045-D10; Aibio, Shanghai, China) as recommended, and imaging was conducted using the ChemiDoc XRS+ system (Bio-Rad).

### Immunofluorescent assay to evaluate protein expression in *H. contortus*

Purified polyclonal antibody against r*Hco-*DAF-8 was used to detect the expression of native *Hco*-DAF-8 in parasite sections. Briefly, freshly collected *H. contortus* adults were fixed overnight in 4% paraformaldehyde (PFA) in PBS at 4 °C, as described previously [32]. Subsequently, the worms were washed, dehydrated, immersed in paraffin wax and cut into 4 μm-thick sections. Sections were processed and incubated in 3% hydrogen peroxide for 10 min at room temperature (24 °C) to quench endogenous peroxidase activity. The sections were then pre-blocked with bovine serum albumin (BSA) for 20 min at 37 °C, before probing overnight with the anti-r*Hco-*DAF-8 antibody (1:100 in PBST) at 4 °C. Negative control sections were probed with the pre-immune rabbit serum (1:100 dilution). Following three washes (5 min each), the sections were subjected to incubation with the Alexa Fluor® 594 goat anti-rabbit IgG antibody ReadyProbes® reagent (R37117; Thermo Fisher Scientific) at a 1:3000 dilution for 50 min at 37 °C. Unbound secondary serum was removed before an incubation with 4′,6-diamidino-2-phenylindole (DAPI) for 5 min at 24 °C. Subsequently, worm sections were washed and mounted in mounting-medium [33]. Fluorescence was detected using a fluorescence microscope (BX51; Olympus, Tokyo, Japan).

### Construction of plasmids and gene rescue in *C. elegans*

To conduct the gene rescue experiment in *C. elegans*, two plasmids including a test plasmid *Cel-daf-8p::Hco-daf-8::gfp* and a positive control plasmid *Cel-daf-8p::Cel-daf-8::gfp* were constructed (Additional file 1: Table S1, Additional file 2: Figure S1). In brief, the predicted promoter of *Cel-daf-8* was PCR-amplified from the genomic DNA of *C*. *elegans* using a forward primer Cel-daf-8p-jy-F (Additional file 1: Table S1) containing a linker sequence (21 bp) identical to a partial sequence of pPV199 and a reverse primer Cel-daf-8p-jy-R (Additional file 1: Table S1) employing the following cycling conditions: 98 °C for 5 min; then 98 °C for 10 s, 55 °C for 5 s, 72 °C for 3 min for 35 cycles; followed by 72 °C for 5 min. The full length coding sequence (CDS) of *Hco-daf-8* was PCR-amplified using the primer pair Hco-daf-8-jy-F/Hco-daf-8-jy-R (Additional file 1: Table S1) from *H. contortus* cDNA under the following protocol: 98 °C for 5 min; then 95 °C for 10 s, 55 °C for 5 s, 72 °C for 90 s for 35 cycles; followed by 72 °C for 5 min. The vector pPV199 was linearised by *Age*I and *Bam*HI. Then three fragments were ligated together to generate a test rescue plasmid *Cel-daf-8p::Hco-daf-8::gfp* using CloneExpress® Multis One Step Cloning Kit (Vazyme, Nanjing, China). Similarly, the CDS of *Cel-daf-8* was amplified from the cDNA of *C. elegans* using the primer pair Cel-daf-8-jy-F/Cel-daf-8-jy-R (Additional file 1: Table S1) and then ligated with the amplified promoter sequence of *Cel-daf-8* and the digested pPV199 fragment to produce the positive control plasmid *Cel-daf-8p::Cel-daf-8::gfp* for the rescue assay. The sequences of the inserts of the two plasmids were each verified by sequencing.

The rescue assay was conducted as described previously [20]. Rescue plasmid (50 ng/μl) was micro-injected into the adult germline of *daf-8* (CB1393) mutant worms, together with the pRF-4 (50 ng/μl) as a marker for transformation. The transgenic strain, selected based on its green fluorescence/roller phenotype, was assessed for rescue. For each transgenic line, 15 green fluorescent protein (GFP)-positive, gravid hermaphrodites were transferred to an NGM plate containing OP50 and allowed to lay eggs at 16 °C for 3 h. Then, adults were removed, and eggs were kept at 25 °C for 3 days. Adult and dauer worms with green fluorescence were counted. Dauer was assessed based on the radial constriction of the body and a lack of pharyngeal pumping [7]. The percentage of worms rescued was calculated based on the number of adults with GFP divided by total number of GFP-positive worms. Each phenotypic assay was repeated at least three times per transgenic line. GFP expression of the rescued worms were examined using a compound epifluorescence microscope (BX51; Olympus) with Nomarski differential interference contrast (DIC) optics equipped with a digital camera.

### Inhibitor assay

The specific inhibitor of human Smad3 (SIS3) was purchased from MedChemExpress (HY-13013; Shanghai, China). SIS3 was stored as a solution in dimethyl sulfoxide (DMSO) at a final concentration of 50 mM; this solution was used after being diluted to 0.5 mM with Luria Bertani medium containing 2.5 μg/ml amphotericin, 100 μg/ml streptomycin and 100 IU/ml penicillin (Gibco, New York, USA) (LB*, [34]) for each assay. Seven groups were set up, including a LB* group, negative control group (0 µM SIS3) with maximum DMSO (2.0%), and five experimental groups with different inhibitor concentrations ranging from 2 µM to 50 µM (5 steps). Infective L3s were exsheathed with 0.15% sodium hypochlorite for 30 min at 38 °C, followed by 6 washes in PBS and 3 washes in LB*. Exsheathed L3s (xL3s) were incubated in 100 µl LB* at a concentration of 500–1000 larvae/ml. Inhibitor was added to xL3s at a final concentration of 2 µM to 50 µM in five different experimental groups in triplicate, and larvae were cultured for 7 days at 37 °C under 20% (v/v) CO_2_. The percentages of xL3s that developed to L4s *in vitro* were counted using a microscope (cf. [35]). Experiments were repeated three times.

## Results

### Structural features and sequence analysis of *Hco*-DAF-8

The full-length genomic DNA sequences of *Hco-daf-8* and *Cel-daf-8* were each amplified and sequenced. The sequences of *Hco-daf-8* and *Cel-daf-8* were identical to HF958885.1 and NC_003279.8 (GenBank databases), respectively. Although similar in gene length (~ 3000 bp long) and CDS length (~1500 bp) to *Cel-daf-8*, *Hco-daf-8* has 12 exons (81–221 bp) and 11 introns (50–630 bp) compared with 6 exons (93–477 bp) and 5 introns (123–560 bp) in *Cel-daf-8* (Additional file 3: Figure S2).

**Fig. 1:**
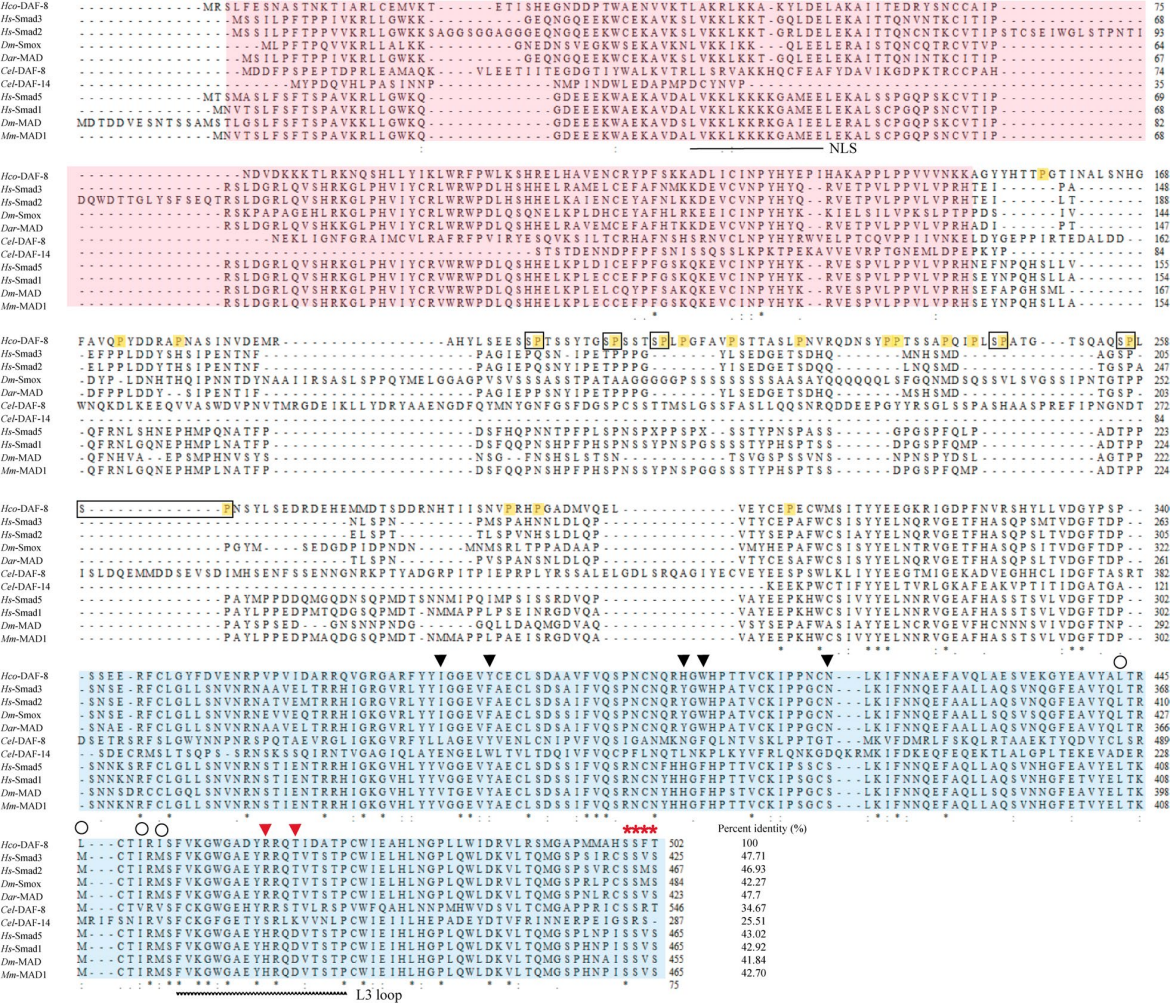
Amino acid sequence alignment of *Haemonchus contortus Hco-*DAF-8 with those of 10 R-Smads from selected species, including one nematode (*Caenorhabditis elegans*), one arthropod (*Drosophila melanogaster*) and three chordates (*Danio rerio, Homo sapiens* and *Mus musculus*). Sequence motifs, such as MH1 (pink), MH2 (blue) and the phosphorylation motif (****) are marked. Within the MH1 region, the predicted nuclear localisation signal (NLS) is underlined. Within the linker region between the MH1 and the MH2 domain of *Hco*-DAF-8, prolines (yellow) and SP motifs (open squares) which function as target sites for phosphorylation *via* intracellular serine/threonine kinases are highlighted. Within the MH2 domain, black arrow indicates the conserved residues involved in TGF-β/Activin-activated R-Smads interaction with SARA; open circle indicates conserved residues of the nuclear export signal. The L3 loop sequence (18 amino acids) is shown (wavy line below the alignment) with R-Smad subtype-specific amino acid residues highlighted with red arrows at the top. The sequence percent identities (%) of *Hco*-DAF-8 with the other R-Smads are listed at the end of the alignment. Information on sequences in the alignment is listed in Additional file 1: Table S2

The full-length open reading frame of *Hco-daf-8* was 1509 bp in length, encoding *Hco*-DAF-8 of 502 aa. The alignment of amino acid sequences indicated that all selected R-Smads have a Mad homology 1 (MH1) domain (pink) at the N-terminus, which binds DNA and a Mad homology 2 domain (MH2, blue) at the C-terminus, which participates in inter-molecular interactions and regulates transcription [36]. In the MH1 domain, a nuclear localisation signal (NLS) sequence LAKRLKKAKYLDE [27] was identified (Fig. 1). Between the MH1 domain and the MH2 domain is a proline-rich ligation region containing six SP motifs (Fig. 1), which function as target sites for phosphorylation by intracellular serine/threonine kinases [37]. Within the MH2 domain, three of the five subtype-specific residues (black arrowhead in Fig. 1) in the MH2 domain of *Hs*-Smads 2 and 3, which are important for the interaction with Smad anchoring for receptor activation (SARA) [38], were identified in *Hco*-DAF-8 (Fig. 1). In addition, also within the MH2 domain, two of the four conserved large, non-polar residues (open circle) in a characteristic spacing of nuclear export signal (NES) in the MH2 domain [39] were present (Fig. 1). Two residues (R and T, red arrowhead in Fig. 1) in the conserved L3 loop within the MH2 domain, which determine the specific interaction between R-Smads and TGF-β type I receptors [40, 41], were also identified (Fig. 1), whereas BMP-activated R-Smads have two different residues (H and D) (Fig. 1). At the extreme carboxyl terminus of *Hco*-DAF-8, there is a variant SSXT (X represents any amino acid) motif (SSFT) which is similar to that of *Cel-*DAF-8 (SSRT) (Fig. 1), but distinct from the typical SSXS motif of R-Smad, having been demonstrated to be required for the phosphorylation by TGF-β type I receptors [1]. The amino acid sequence comparison showed that *Hco*-DAF-8 has higher identities to the TGF-β/Activin-activated R-Smads including *Hs*-Smad3 (47.7%) and *Hs*-Smad2 (46.9%) than to the BMP-activated R-Smads including *Hs*-Smad1 (42.9%) and *Hs*-Smad5 (43.0%) (Fig. 1). Taken together, these findings suggest that *Hco*-DAF-8 is a member of R-Smads subfamily and has more sequence features in common with TGF-β/Activin-activated R-Smads [4].

### Genetic relationships of *Hco*-DAF-8 with Smads from other species

**Fig. 2:**
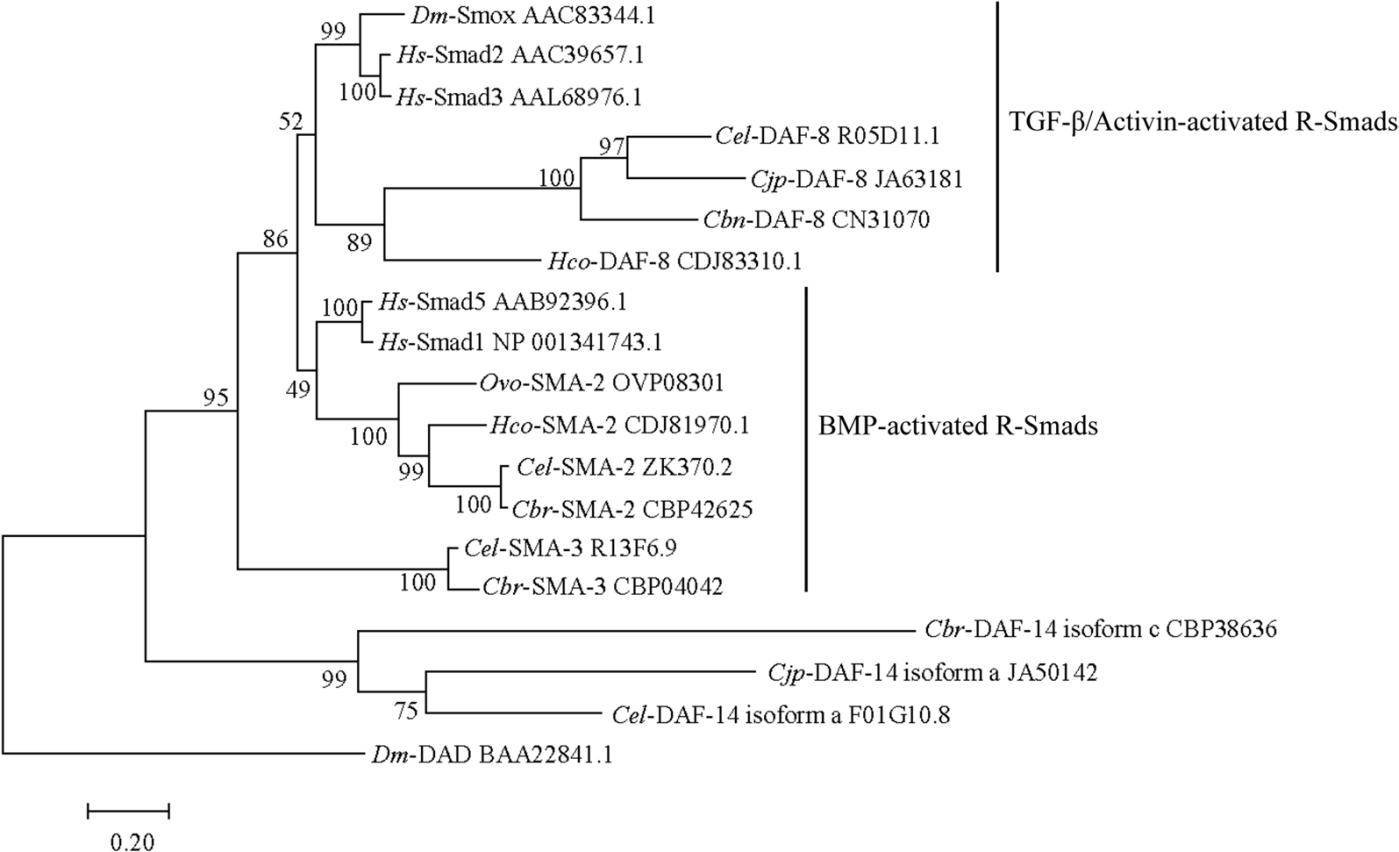
Phylogenetic relationships of *Haemonchus contortus Hco-*DAF-8 with other R-Smads from selected species. The phylogenetic tree was constructed using amino acid sequences of *Hco-DAF-8* and the R-Smad homologues from species, including six nematodes (*Caenorhabditis brenneri*, *C. briggsae*, *C. elegans*, *C. japonica* and *Onchocerca volvulus*), one arthropod (*Drosophila melanogaster*) and one chordate (*Homo sapiens*).The tree was constructed using the neighbour-joining (NJ) method, employing the *Dm*-DAD from *D. melanogaster* as an outgroup. Nodal support values are shown above or below the branches. Accession numbers are listed next to the R-Smad designation. Information on sequences used in the analysis is listed in Additional file 1: Table S2

There are four R-Smads in *C. elegans*, including *Cel-*DAF-8 and *Cel-*DAF-14 which are involved in DAF-7 signalling pathway and *Cel-*SMA-2 and *Cel-*SMA-3 which are involved in the DBL-1 (Dpp and BMP-like-1) signalling pathway. Herein, the aa sequence of *Hco-*DAF-8 was aligned with these four R-Smads and eight homologues of nematodes (*Cjp*-DAF-8, *Cbn*-DAF-8, *Ovo*-SMA-2, *Hco*-SMA-2, *Cbr*-SMA-2, *Cbr*-SMA-3, *Cbr-*DAF-14 and *Cjp*-DAF-14) as well as five R-Smads from *H. sapiens* and *D. melanogaster* (*Hs*-Smad3, *Hs*-smad2, *Hs*-Smad5, *Hs*-smad1 and *Dm*-Smox) for subsequent phylogenetic analysis. Consistent tree topologies were obtained for three analyses (NJ, MP and ML). The results revealed that *Hco*-DAF-8 clustered with three DAF-8s from *Caenorhabditis* species with a nodal support of 89%. In addition, this cluster grouped with three TGF-β/Activin-activated R-Smads of *H. sapiens* and *D. melanogaster* with a nodal support of 52%. On the other hand, four nematode SMA-2s formed a cluster with strong (100%) nodal support. Additionally, two BMP/Dpp-activated R-Smads from *H. sapiens* (*Hs*-Smad1 and *Hs*-Smad5) and two SMA-3s from *Caenorhabditis* species (*Cel-*SMA-3 and *Cbr*-SMA-3) grouped together, with absolute nodal support. The five small clusters (with bootstrap supports ranging from 89 to 100%) grouped together with 95% support to the exclusion of the cluster formed by three DAF-14 (*Cbr*-DAF-14, *Cjp*-DAF-14 and *Cel-*DAF-14) (Fig. 2).

### Transcription of *Hco-daf-8* in different developmental stages of *H. contortus*

**Fig. 3:**
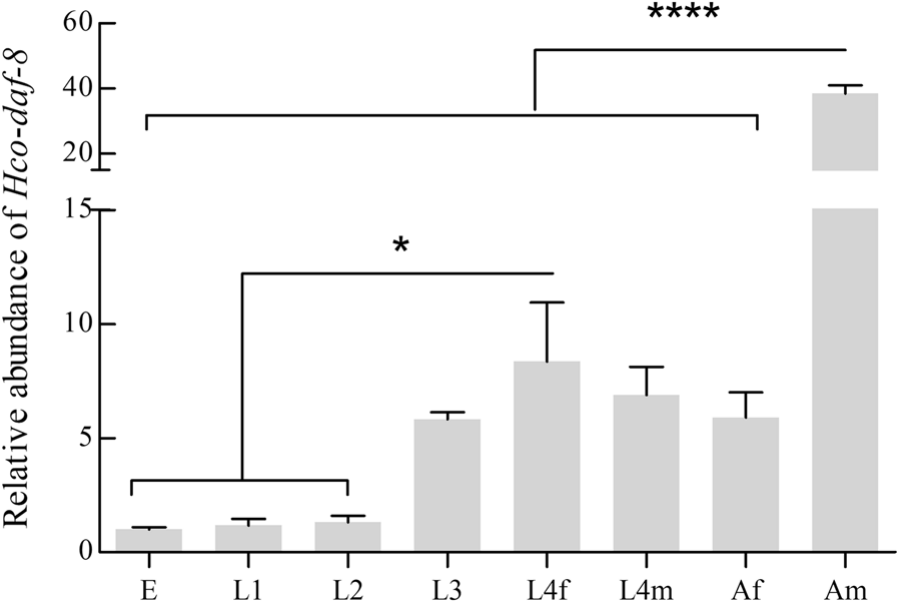
Transcriptional levels of *Hco-daf*-*8* in eight developmental stages/sexes of *Haemonchus contortus.* The relative abundance of *Hco-daf-8* was assessed in eight developmental stages of *H. contortus*: eggs (E); first-stage larvae (L1); second-stage larvae (L2); third-stage larvae (L3); fourth-stage females (L4f); fourth-stage males (L4m); adult females (Af); adult males (Am). The relative quantities (compared with egg, egg = 1) are shown as the mean values (± standard error of the mean, SE) derived from three replicates in repeat experiments. All gene transcription levels were normalised against that of the β-tubulin gene. Statistical analysis was carried out using one-way ANOVA. *P*-values are calculated using Tukey’s *post-hoc* test. Significant differences between stages are marked *P < 0.05, ****P < 0.0001

Real-time PCR analysis revealed that *Hco-daf-8* was transcribed in all eight developmental stages/sexes of *H. contortus*, with the lowest levels in the free-living stages and the highest level in the adult male stage, followed by the L4 female stage. The transcript abundance in adult males was markedly higher than that of any other stage (*F*_(7, 16)_ = 77.48, *P* < 0.0001). In addition, there was a significant difference between L4 female and each egg (*P* = 0.03), L1 (*P* = 0.04) and L2 (*P* = 0.04) stages. No significant difference was observed between other stages (Fig. 3).

### Expression of *Hco*-DAF-8 in *H. contortus* by immunofluorescence

**Fig. 4:**
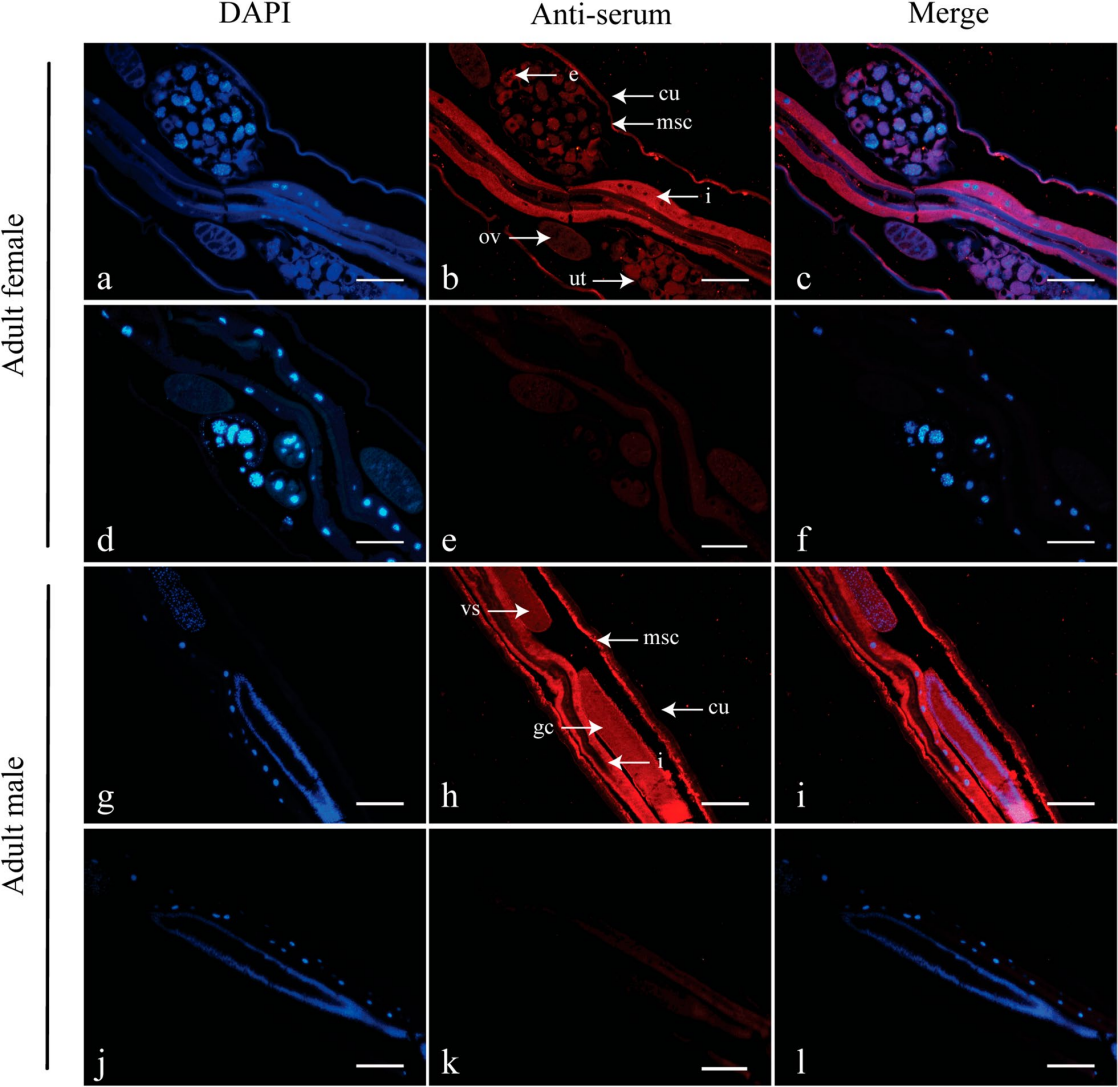
The localization of *Hco-*DAF-8 in *Haemonchus contortus* adults by immunofluorescence. **a**–**c** Localization of *Hco-*DAF-8 in *H. contortus* adult females: in the platymyrian muscle cells (msc) under cuticle, in intestine (i) and some eggs (e). **g**–**i** Localization of *Hco-*DAF-8 in adult males: in the platymyrian muscle cells (msc) under cuticle, in intestine (i), seminal vesicle (vs) and cement gland (gc). Ovaries (ov), cuticle (cu) and uterus (ut) are also indicated. No fluorescence labelling was observed in negative controls probed with serum representing the pre-bleed prior to immunization (**d**–**f, j**–**l**). DAPI (4′,6-diamidino-2-phenylindole, dihydrochloride) is used to label the nucleus. *Scale-bars*: 100 μm

The cDNA encoding partial *Hco-*DAF-8 (251 aa) was expressed in *E. coli* with an expected size of 53 kDa (Additional file 4: Figure S3a), and the purified recombinant protein was used to immunise rabbits to produce serum antibody. The polyclonal antibody (positive-serum) from rabbits bound specifically to the native *Hco*-DAF-8 (~ 56.5 kDa) from both sexes of *H. contortus* adults, while the pre-immune serum did not recognize any proteins in *H. contortus* (Additional file 4: Figure S3b). Using this antibody as a probe, *Hco*-DAF-8 was shown to be highly expressed in the platymyrian muscle cells under the cuticle and the intestine in both female and male worms. In addition, it was also expressed in some eggs in the female as well as the seminal vesicle and cement gland in the male (Fig. 4a–c, g–i). There was no labelling in worm sections probed with pre-immune rabbit serum (Fig. 4d–f, j–l).

### Genetic rescue of the *daf-8* deficient *C. elegans* with *Hco-daf-8*

**Fig. 5:**
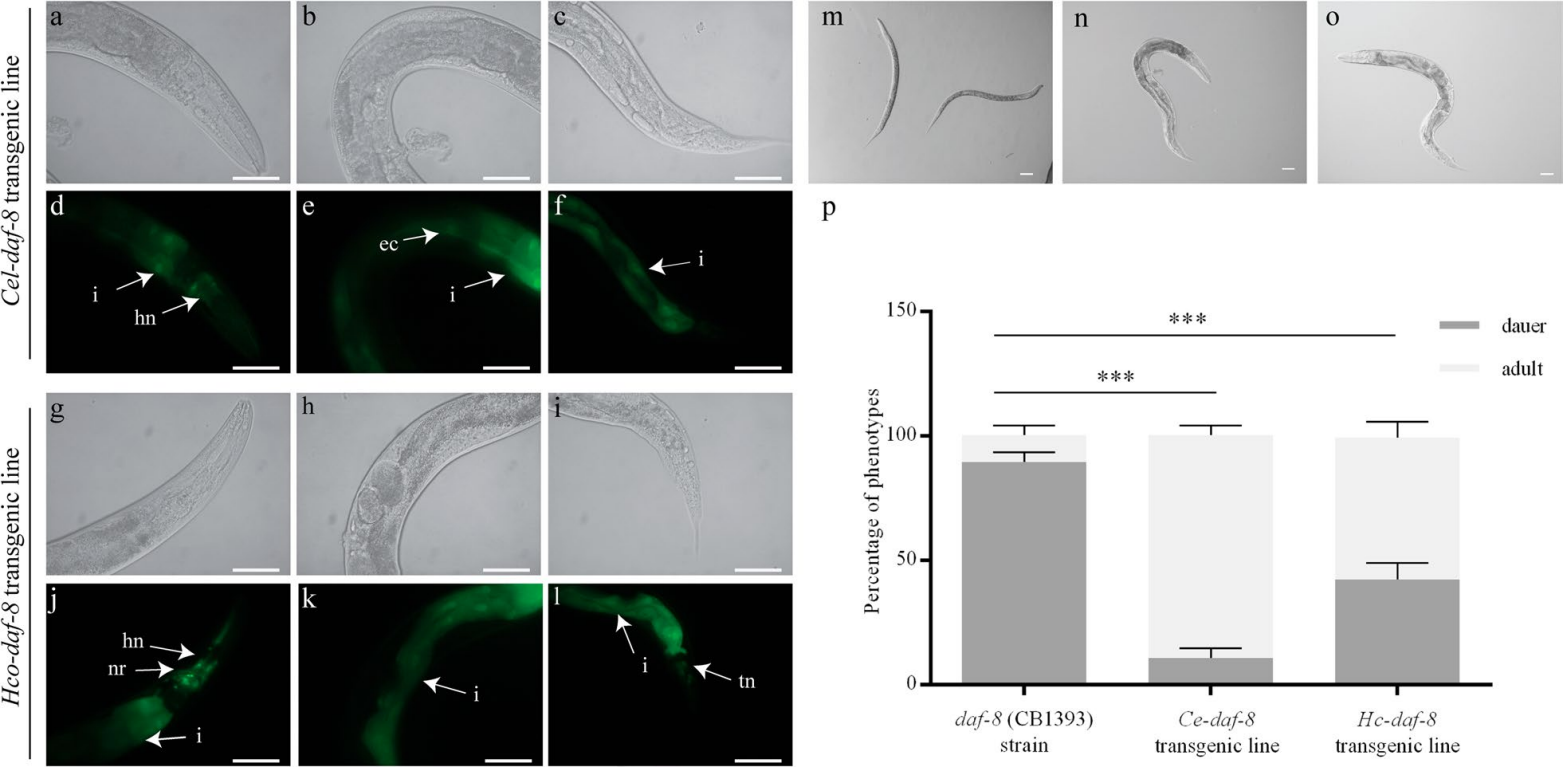
Results of gene rescue assays on mutant and transgenic *Caenorhaboditis elegans* strains. **a**–**f** The anatomical expression pattern in transgenic *C. elegans* strain transformed with *Cel-daf-8p::Cel-daf-8::gfp.***g**–**l** The anatomical expression pattern in transgenic *C. elegans* strain transformed with *Cel-daf-8p::Hco-daf-8::gfp.***a**–**c**, **g**–**i** are the DIC images of the rescued worms. **d**–**f**, **j**–**l** are the representative GFP expression patterns. Intense green fluorescent signals were detected in the intestine (i) head neurons (hn) nerve-ring (nr), excretory duct cells (ec) and tail neurons (tn). **m** Non-rescued mutant worms remain at the dauer stage at 25 °C with constrictive body and stationary pharynx. **n**, **o** Worms that developed to adult worms rescued successfully with the plasmids *Cel-daf-8p::Cel-daf-8::gfp* or *Cel-daf-8p::Hco-daf-8::gfp* at 25 °C, respectively. **p** Percentage of different phenotypes of worms that developed at 25 °C in the *daf-8 C. elegans* mutant strain (CB1393) and the *C. elegans* transgenic strains. ***P < 0.001. *Scale-bars*: 50 μm

The *Cel-daf-8* mutant strain (CB1393) of *C. elegans* is temperature-sensitive, and can normally develop to the adult stage at 16 °C but remains as a dauer stage at 25 °C. To assess whether *Hco-daf-8* could rescue the *daf-8* deficient *C. elegans* (CB1393) mutant strain, two transgenic *C. elegans* lines were constructed by micro-injecting plasmid *Cel-daf-8p::Cel-daf-8::gfp* (positive control) or *Cel-daf-8p::Hco-daf-8::gfp* (test plasmid) into the *Cel-daf-8* mutant strain. The results showed that *Cel-daf-8p::Hco-daf-8::gfp* was expressed in all life stages, and GFP expression was mainly detected in the intestine and nervous system (Fig. 5g–l), consistent with the expression patterns in worms transformed with *Cel-daf-8p::Cel-daf-8::gfp* (Fig. 5a–f). In *C. elegans* control-transgenic lines transformed with *Cel-daf-8p::Cel-daf-8::gfp* plasmid, ~ 90% (89.5 ± 4.0%) of the offspring expressing GFP developed to adults (Fig. 5n, p). In contrast, the transgenic lines transformed with *Cel-daf-8p::Hco-daf-8::gfp* plasmid, partial (57 ± 6.4%) offspring developed to adults (Fig. 5o, p). The pharynx in rescued worms resumed pumping, the vulva projected and the uterus contained eggs, while ‘unrescued’ worms remained as dauer after the same period of development (Fig. 5m). There were significant differences in the percentages of developed adult worms in *Ce-daf-8* transgenic line and *Hc-daf-8* transgenic line compared with the untreated groups (*F*_(2, 9)_ = 65.58, *P* < 0.0001 and *P* = 0.0002, respectively) (Fig. 5p).

### SIS3 inhibits the development of *H. contortus* from xL3s to L4s

**Fig. 6:**
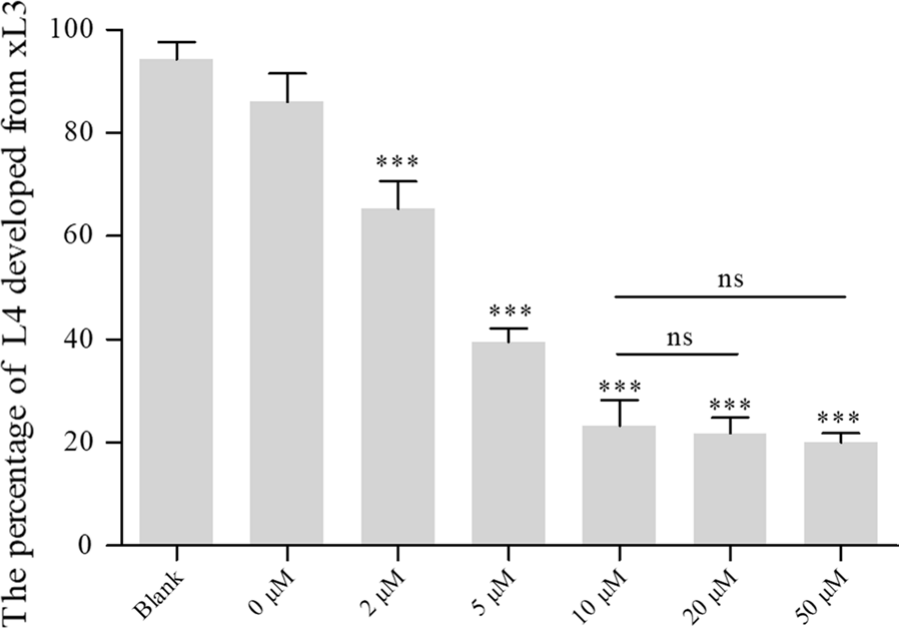
The effect of specific inhibitor of human Smad3 (SIS3) on the development of *Haemonchus contortus*. Seven groups were set up including LB* blank control group, DMSO group with maximum concentration (2%) as a negative control, and experimental groups of different inhibitor concentrations from 2 µM to 50 µM (5 steps). The percentages of L4s that developed from xL3s *in vitro* were counted. Experiments were repeated three times. Significant difference compared with the blank and DMSO (negative) groups are marked ***P < 0.001; ns, no significant difference

To further assess the functionality of *Hco*-DAF-8 as an R-Smad protein, the inhibitor assay using R-Smad inhibitor SIS3 was conducted *in vitro*. The xL3s of *H. contortus* developed to L4s *in vitro* under normal culture conditions. Compared with xL3s, the buccal capsule of L4s was fully developed and functional [42, 43]. Results showed that SIS3 significantly inhibited L4 development at five concentrations from as low as 2 µM to 50 µM (5 µM, 10 µM, 20 µM and 50 µM) (*F*_(6, 21)_ = 60.45, *P* < 0.001). The inhibitory effect was dose-dependent, with an increased inhibition in the concentration range of 2 µM to 10 µM, reaching a maximum inhibitory effect at 10 µM and beyond (Fig. 6). There were no significant differences among groups 10 µM, 20 µM and 50 µM (*F*_(2, 9)_ = 0.21).

## Discussion

In the present study, an R-Smad protein coding gene (*Hco-daf-8*) and its predicted product (*Hco*-DAF-8) of *H. contortus* were identified and characterised both structurally and functionally. *Hco*-DAF-8 has characteristics consistent with TGF-β/Activin-activated R-Smads, especially the two distinctive amino acids (R^462^ and T^465^) in the L3 loop determining the specific interaction between R-Smads and TGF-β type I receptors [40, 41]. In addition, phylogenetic analysis showed that *Hco-*DAF-8 is closely related to DAF-8 homologues of *Caenorhabditis* species and grouped with the TGF-β/Activin-activated R-Smads. These results suggest that *Hco*-DAF-8 is a member of TGF-β/Activin-activated R-Smads that usually transmit the TGF-β signals.

To understand at which time points *Hco*-DAF-8 functions in *H. contortus*, the transcription of its encoding gene *Hco-daf-8* in different developmental stages of *H. controtus* was assessed by real-time PCR. *Hco-daf-8* was transcribed in all developmental stages studied, with the lowest transcription in the egg, L1 and L2 (free-living) stages, higher transcription in L4 females, and the highest level in adult males. In contrast, *C. elegans daf-8* transcript level peaked in the early larval stages (egg and L1 stages), but then declined to low levels in L2 to adult stages [44]. This finding indicates that *Cel-daf-8* might play a role in mediating developmental switch (entry into dauer or normal L3 stage) in *C. elegans*, while *Hco-daf-8* may influence the continuous development of *H. contortus* from iL3s as larval arrest is facultative in *C. elegans*, but constitutive in the parasitic nematodes [45, 46]. Recent findings from RNAi-based studies also confirm functional roles for genes encoding TGF-β type I receptor (*Hc-tgfbr1*) [12], TGF-β type II receptor (*Hc-tgfbr2*) [13] and co-Smad (*Hc-daf-3*) [14] in the development from the free-living to the parasitic stages of *H. contortus*.

The high transcript level of *Hco*-DAF-8 and notable protein expression in the male reproductive system may indicate an important role of this molecule in male *H. contortus*. In *C. elegans*, *Cel-*DAF-8 was proven to negatively regulate the germline proliferative zone when it was expressed in the distal tip cell [10]. Another study [47] showed that a reduction of the TGF-β Sma/Mab signalling pathway in *C. elegans* extends the reproductive span by maintaining oocyte and germline quality. In addition, in the flatworm *Schistosoma mansoni*, it has been shown that TGF-β signalling pathway is crucial for the embryo development and egg production [48, 49]. We speculate that *Hco*-DAF-8 is involved in parasite growth and development as well as germline quality maintenance and reproduction. The expression of eggs in the female uterus indicates a possible effect of this molecule on the embryogenesis and development. Interestingly, the high level of transcription in adult males is also seen for *Hc-tgftr2* [13]; both *Hco*-DAF-8 and *Hc*-TGFTR2 are highly expressed in the reproductive system. The similar transcriptional and expression patterns of these two molecules suggest that the *Hco-*DAF-8 may function together with *Hc*-TGFTR2 in reproductive development in *H. contortus*.

Besides the expression in the reproductive system, *Hco-*DAF-8 was also highly expressed in the intestinal cytoplasm of *H. contortus* adult worms, indicating that this protein might affect the digestive system and/or be immunogenic, given this worm’s gut is a source of effective vaccine molecules [50]. Its expression in the platymyrian muscle cells under cuticle suggests that *Hco-*DAF-8 or TGF-β signalling pathway may relate to cuticle formation, muscle movement, the transmission of external signals from host and ionic homeostasis [51]. Collectively, these findings might indicate an important role for *Hco*-DAF-8 in growth and development and/or as a regulator of transcription in response to developmental cues.

To verify whether the function of *Hco-daf-8* is similar to that of *Cel-daf-8*, gene rescue was performed. Results showed that *Hco-daf*-*8* can be expressed in the specific tissues consistent with *Cel-daf-8* and can rescue the dauer phenotype of *Cel-daf-8* mutant strain but with less efficiency. This is possibly due to differences in the target gene sequence and the transgene expression efficiency. Other reasons could be species-specific differences in gene expression that might have been reflected in suboptimal expression/function of *H. contortus* elements in *C. elegans.* Moreover, *Cel-*DAF-8 encoded by *Cel-daf-8* interacts with some other endogenous proteins like *Cel-*DAF-14 [10], although it is possible that the binding of *Hco-daf-8* to endogenous *Cel-daf-14* is not as strong as to *Cel-daf-8* in *C. elegans.* Nevertheless, the effective rescue indicated that the biochemical properties of *Hco*-DAF-8 are similar enough to those of *Cel-*DAF-8, such that it can function as *Cel-*DAF-8 when expressed at a corresponding location and time in *C. elegans.*

It is well known that *Cel-daf-8* regulates dauer formation *via* TGF-β signalling. Mutant *C. elegans* rescued by *Hco*-DAF-8 can restore TGF-β signalling to complete normal development. To answer as to whether *Hco-daf-8* participates in this signalling pathway and plays key roles in the development of *H. contortus* L3 (analogous to dauer in *C. elegans*), we used a Smad3-specific inhibitor (SIS3) to assess TGF-β signalling pathway function. SIS3 was first reported as a potent and selective inhibitor of Smad3 in this pathway of *H. sapiens* and affected neither the phosphorylation of Smad2 nor the phosphorylation of other signalling pathways [52]. Due to its important functions in suppressing cancer growth, invasion and metastasis, and in preventing the cancer death, SIS3 was described in a patent as a novel and effective anti-cancer drug by way of regulating cellular signal transduction mediated by TGF-β/Smad3 in the USA [53]. Moreover, recently, SIS3 was applied to the parasitic flatworm *Echinococcus granulosus*, resulting in a marked effect on the key components of the Smad signalling pathway, inhibiting the growth and survival of protoscoleces [54]. These published findings stimulated our interest in assessing this inhibitor on *H. contortus in vitro*. The results revealed that SIS3 inhibited the development of L3 larvae in a dose-dependent manner, suggesting a role for the Smad3 homologue in larval development in *H. contortus*, like other members of the TGF-β signalling pathway studied previously in this parasite [12–14]. Sequence analysis showed that *Hco-*DAF-8 has a higher similarity with *Hs*-Smad3 than the other Smads in *H. sapiens* at the amino acid level, suggesting that SIS3 specifically inhibits the function of *Hco*-DAF-8 in *H. contortus*. Future work might conduct structure activity relationship (SAR) studies of SIS3 analogs to establish whether they might show promise as a new nematocide for *H. contortus* and related worms.

In *C. elegans*, *Cel*-DAF-8 can inhibit the Co-Smad *Cel*-DAF-3 or *vice versa*. Herein, overlapping expression can be detected in the platymyrian muscle cells under the cuticle in both sexes of adult worms or in the cement gland in the adult male between *Hco*-DAF-3 and *Hco*-DAF-8[14], suggesting some common roles in these tissues. Considering that chemical inhibition resulted in the retarded larval development *in vitro*, we speculate that R-Smads positively regulate the downstream Co-Smads in the cuticle, which sense the host environment and promote transition from the free-living stage (L3s) to the parasitic stage (L4s) in *H. contortus*.

## Conclusions

We identified a gene encoding an R-Smad protein (*Hco*-DAF-8) in *H. contortus*. *Hco-*DAF-8 is an R-Smad with inferred functional domains typical of the Smad protein family. This protein is widely expressed and appears to control the development of xL3s to L4s in *H. contortus.* The highest transcription level and high protein expression in the reproductive organs of male adults suggest that *Hco*-DAF-8 has a role in male reproduction. SIS3 inhibited the development from the xL3 to the L4 stage, suggesting a key role in developmental transition. Taken together, the findings of this study demonstrate that *Hco*-DAF-8 is a key molecule in the TGF-β signalling pathway of *H. contortus* and regulates development and/or reproduction in this worm.

## Supplementary information

**Additional file 1: Table S1.** Oligonucleotide primers used in the present study. **Table S2.** Sequences used for phylogenetic and alignment analyses.

**Additional file 2: Figure S1.** Schematic diagram explaining the process of constructing gene rescuing plasmids by homologous recombination. The *C. elegans Cel-daf-8* promoter region was PCR-amplified using primers F1/R1. In addition, the coding region (CDS) of *Cel-daf-8* or *H. contortus Hco-daf-8* was amplified using primers F2/R2 or F3/R3, respectively. Besides, the GFP reporter vector pPV199 was digested simultaneously with the restriction enzymes *Bam*HI and *Age*I. Then, the three fragments (including *Cel-daf-8* promoter, *Cel-daf-8* CDS and the digested pPV199 or *Cel*-*daf-8* promoter, *Hco*-*daf-8* CDS and the digested pPV199) were used together for homologous recombination *in vitro* to produce the rescuing plasmid (*Cel-daf-8p::Cel-daf-8::gfp* or *Cel-daf-8p::Hco-daf-8::gfp*). Primer sequences used here are listed in Additional file 1: Table S1.

**Additional file 3: Figure S2.** Gene structures of R-Smad homologues from *H. contortus* (*Hco-daf*-*8*) and *C. elegans* (*Cel-daf-8*). Black boxes represent exons and the numbers above display the lengths of exons. Lines between the exons represent introns, and the numbers below indicate the lengths of introns.

**Additional file 4: Figure S3.** Expression and purification of recombinant *Hco*-DAF-8 protein of *H. contortus* and immunoblot analysis. **a** Prokaryotic expression of recombinant protein *Hco*-DAF-8. Lane M: protein marker; Lane 1: expressed products of pGEX-4T-*Hco-*DAF-8 (94–344) non-induced; Lane 2: expressed products of pGEX-4T-*Hco-*DAF-8 (94–344) induced; Lane 3: expressed products of pGEX-4T empty vector induced; Lane 4: purified pGEX-4T-*Hco-*DAF-8 (94–344) protein. **b** Immunoblot analysis of natural *Hco*-DAF-8 protein. Lane M: protein marker; native *Hco*-DAF-8 protein was detected in adult male and female using anti-r*Hco*-DAF-8 anti-serum (antiserum), using a pre-immune serum as a control.

## Data Availability

Data supporting the conclusions of this article are included within the article and its additional files. The amino acid sequence of *Hco*-DAF-8 is available on GenBank under the accession number CDJ83310.1.

## Abbreviations

TGF-β: transforming growth factor-β
R-Smad: receptor-regulated Smad
SIS3: specific inhibitor of human Smad3
Co-Smads: common-partner Smads
I-Smads: inhibitory Smads
NGM: nematode growth medium
EBSS: Earle’s balanced salt solution
CDS: coding DNA sequence
r*Hco-*DAF-8: recombinant *Hco-*DAF-8
IPTG: isopropyl β-D-1-thiogalactopyranoside
SDS: sodium dodecyl sulfate
PBS: phosphate-buffered saline
PFA: paraformaldehyde
DAPI: 4′, 6-diamidino-2-phenylindole
GFP: green fluorescent protein
DMSO: dimethyl sulfoxide
SARA: Smad-anchoring for receptor activation
NLS: nuclear localisation signal
NES: nuclear export signal
DBL-1: Dpp and BMP-like-1
SAR: structure activity relationship
