# Supplementary material for: Identification and characterization of an R-Smad homologue (Hco-DAF-8) from Haemonchus contortus
Source: Parasit Vectors. 2020 Apr 3;13:164. doi: 10.1186/s13071-020-04034-0 (PMC7119156; doi:10.1186/s13071-020-04034-0)
Supplement: Supplementary file 1 — Additional file 1: Table S1. Oligonucleotide primers used in the present study. Table S2. Sequences used for phylogenetic and alignment analyses. [file 13071_2020_4034_MOESM1_ESM.docx]

**Additional file 1: Table S1** Oligonucleotide primers used in the present study.

| **Primer** | **Sequence (5’-3’)** |
| --- | --- |
| *For amplification of CDS* | |
| Hco-daf-8-F | ATGCGATCCTTGTTCGAA |
| Hco-daf-8-R | CTACGTAAATGAGGAA |
| *For construction of expression plasmid* | |
| rHco-DAF-8-F | CGCGGATCCATAAAATTGTGGAGATTTCCATGGC |
| rHco-DAF-8-R | CCGCTCGAGCTCTTCAGAACTTGGAGAAGGGTAA |
| *For construction of gene rescue plasmids* | |
| Cel-daf-8p-jy-F (F1) | *TGCAGGTCGACTAGAGGATCC*GTGCCAGCATCGCGCGCACATTTTG |
| Cel-daf-8p-jy-R (R1) | GGTGAAGGAAAATCGTCCAT |
| Hco-daf-8-jy-F (F2) | *TCAGTTTTAAAACTACGAAA*ATGCGATCCTTGTTCGAATCG |
| Hco-daf-8-jy-R (R2) | *TTACTCATTTTTTCTACCGGT*TTCGTAGTTTTAAAACTGA |
| Cel-daf-8-jy-F (F3) | *TCAGTTTTAAAACTACGAAA*ATGGACGATTTTCCTTCACC |
| Cel-daf-8-jy-R (R3) | *TTACTCATTTTTTCTACCGGT*GTTCTGGATGAACATATAC |
| *For real-time PCR* | |
| rtHco-daf-8-F | ATGCGCTCAGCAATCATGGA |
| rtHco-daf-8-R | GTCGGGGAACTCTCTTCGGA |
| Tubulin-F | TGTTCCATCACCCAAGGTATCC |
| Tubulin-R | TGACAGACACAAGGTGGTTGAGAT |

Italics represents homologous sequences from vector or specific gene and underscore represents the restriction site. The F1-F3 and R1-R3 as brief description are corresponding to the primers used for constructing gene rescuing plasmids shown in Additional file 2: Figure S1.

**Additional file 1: Table S2.** Sequences of R-Smads used for phylogenetic and alignment analyses.

| **Species** | **Protein name** | **GenBank/WormBase**  **accession number** |
| --- | --- | --- |
| *Caenorhabditis brenneri* | *Cbn*-DAF-8^b^ | CN31070^e^ |
| *Caenorhabditis brenneri* | *Cbr*-DAF-14 isoform c^b^ | CBP38636^e^ |
| *Caenorhabditis briggsae* | *Cbr*-SMA-3^b^ | CBP04042^e^ |
| *Caenorhabditis briggsae* | *Cbr*-SMA-2^b^ | CBP42625^e^ |
| *Caenorhabditis elegans* | SMA-2^b^ | ZK370.2^e^ |
| *Caenorhabditis elegans* | SMA-3^b^ | R13F6.9^e^ |
| *Caenorhabditis elegans* | DAF-14^a,b^ | F01G10.8^e^ |
| *Caenorhabditis elegans* | DAF-8^a,b^ | R05D11.1^e^ |
| *Caenorhabditis japonica* | *Cjp*-DAF-8^b^ | JA63181^e^ |
| *Caenorhabditis japonica* | *Cjp*-DAF-14 isoform a^b^ | JA50142^e^ |
| *Danio rerio* | MAD mothers against decapentaplegic homolog 3b^a^ | AAH81628.1 |
| *Drosophila melanogaster* | MAD polypeptide^a^ | AAB60230.1 |
| *Drosophila melanogaster* | DAD polypeptide^b^ | BAA22841.1 |
| *Drosophila melanogaster* | Smox^a,b^ | AAC83344.1 |
| *Haemonchus contortus* | MAD homology 1 and SMAD domain containing protein^a,b,^ | CDJ83310.1^c^ |
| *Haemonchus contortus* | MAD homology 1 and SMAD domain containing protein^b,^ | CDJ81970.1^d^ |
| *Homo sapiens* | Smad5^a,b^ | AAB92396.1 |
| *Homo sapiens* | Smad1^a,b^ | NP_001341743.1 |
| *Homo sapiens* | Smad2^a,b^ | AAC39657.1 |
| *Homo sapiens* | Smad3^a,b^ | AAL68976.1 |
| *Mus musculus* | mothers against decapentaplegic homolog 1^a^ | NP_032565.2 |
| *Onchocerca volvulus* | *Ovo*-SMA-2^b^ | OVP08301^e^ |

^a^ Sequences were used for alignment analysis.

^b^ Sequences were used for phylogenetic analysis.

^c^ named *Hco*-DAF-8 in this article.

^d^ named *Hco*-SMA-2 in this article.

^e^ WormBase accession number (Version: WS271).
