# Supplementary figures and images for: Identification and characterization of an R-Smad homologue (Hco-DAF-8) from Haemonchus contortus
Source: Parasit Vectors. 2020 Apr 3;13:164. doi: 10.1186/s13071-020-04034-0 (PMC7119156; doi:10.1186/s13071-020-04034-0)

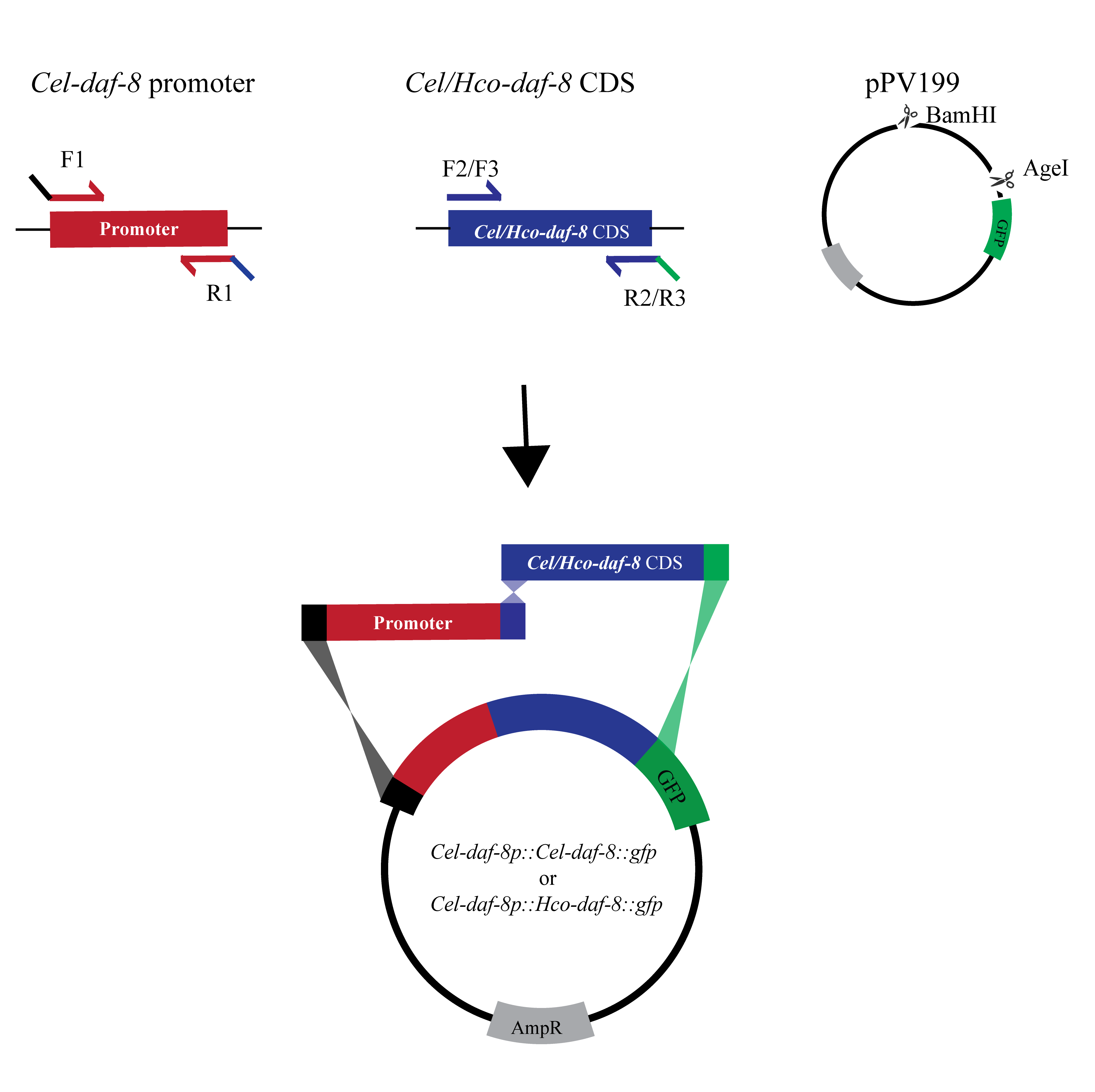

Supplement: Supplementary file 2 — Additional file 2: Figure S1. Schematic diagram explaining the process of constructing gene rescuing plasmids by homologous recombination. The C. elegans Cel-daf-8 promoter region was PCR-amplified using primers F1/R1. In addition, the coding region (CDS) of Cel-daf-8 or H. contortus Hco-daf-8 was amplified using primers F2/R2 or F3/R3, respectively. Besides, the GFP reporter vector pPV199 was digested simultaneously with the restriction enzymes BamHI and AgeI. Then, the three fragments (including Cel-daf-8 promoter, Cel-daf-8 CDS and the digested pPV199 or Cel-daf-8 promoter, Hco-daf-8 CDS and the digested pPV199) were used together for homologous recombination in vitro to produce the rescuing plasmid (Cel-daf-8p::Cel-daf-8::gfp or Cel-daf-8p::Hco-daf-8::gfp). Primer sequences used here are listed in Additional file 1: Table S1. [file 13071_2020_4034_MOESM2_ESM.tif]

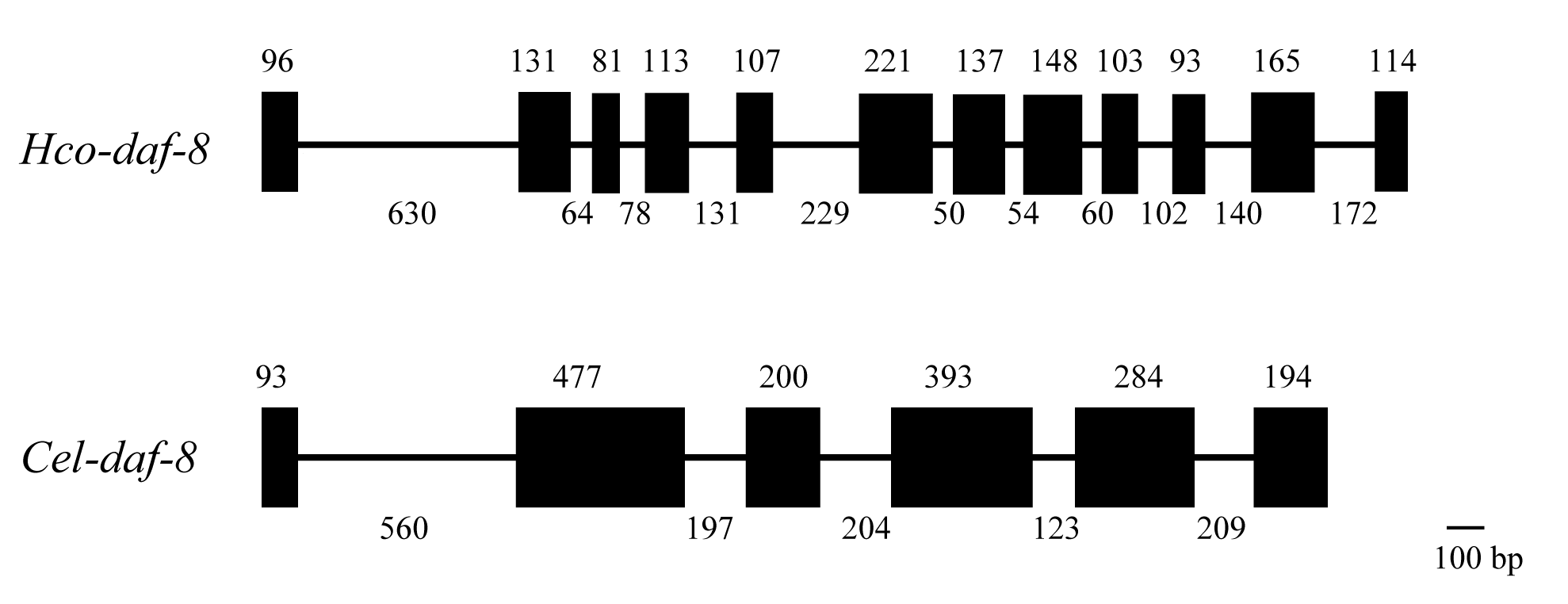

Supplement: Supplementary file 3 — Additional file 3: Figure S2. Gene structures of R-Smad homologues from H. contortus (Hco-daf-8) and C. elegans (Cel-daf-8). Black boxes represent exons and the numbers above display the lengths of exons. Lines between the exons represent introns, and the numbers below indicate the lengths of introns. [file 13071_2020_4034_MOESM3_ESM.tif]

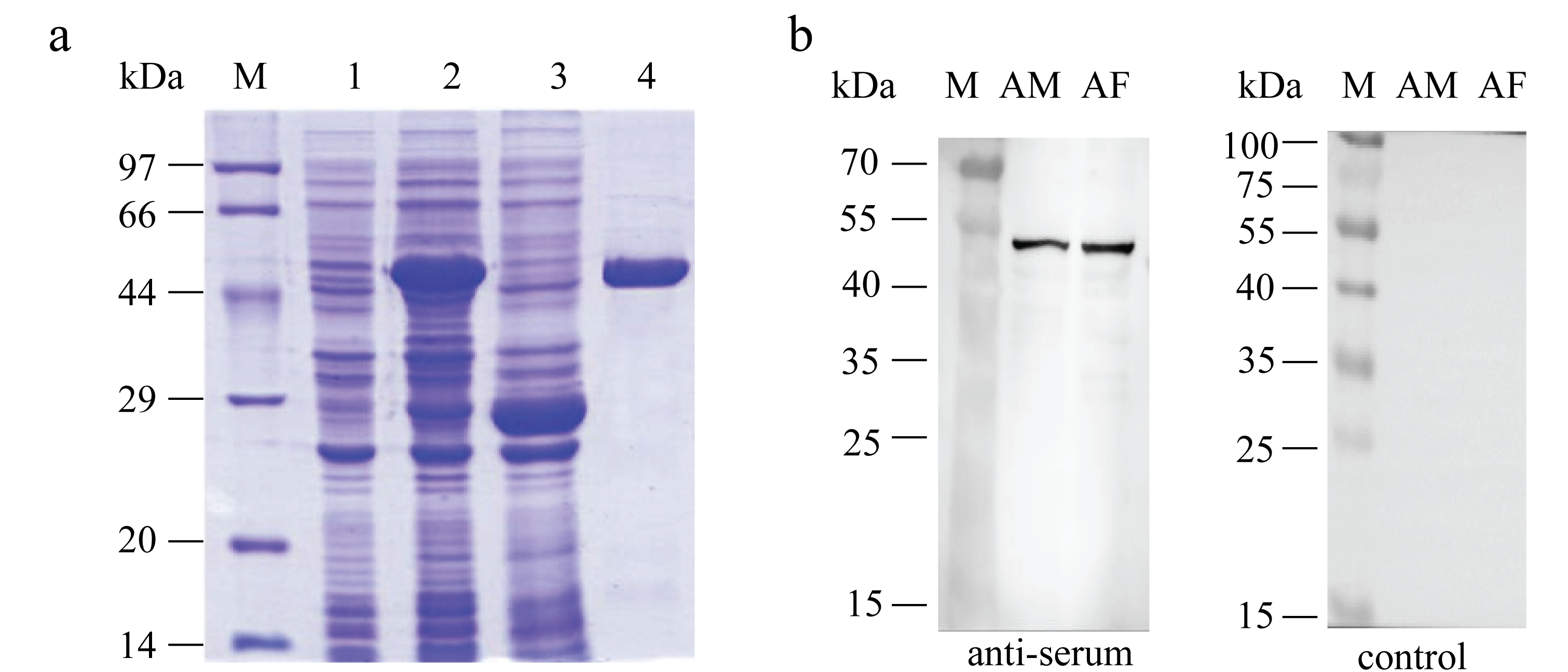

Supplement: Supplementary file 4 — Additional file 4: Figure S3. Expression and purification of recombinant Hco-DAF-8 protein of H. contortus and immunoblot analysis. a Prokaryotic expression of recombinant protein Hco-DAF-8. Lane M: protein marker; Lane 1: expressed products of pGEX-4T-Hco-DAF-8 (94–344) non-induced; Lane 2: expressed products of pGEX-4T-Hco-DAF-8 (94–344) induced; Lane 3: expressed products of pGEX-4T empty vector induced; Lane 4: purified pGEX-4T-Hco-DAF-8 (94–344) protein. b Immunoblot analysis of natural Hco-DAF-8 protein. Lane M: protein marker; native Hco-DAF-8 protein was detected in adult male and female using anti-rHco-DAF-8 anti-serum (antiserum), using a pre-immune serum as a control. [file 13071_2020_4034_MOESM4_ESM.tif]
